# Is Glycated Hemoglobin A1c Level Associated with Adverse Pregnancy Outcomes of Women Affected by Pre-Gestational Diabetes?

**DOI:** 10.3390/medicina57050461

**Published:** 2021-05-09

**Authors:** Serena Xodo, Ambrogio Pietro Londero, Martina D’Agostin, Alice Novak, Silvia Galasso, Carla Pittini, Giovanni Baccarini, Franco Grimaldi, Lorenza Driul

**Affiliations:** 1 Clinic of Gynecology and Obstetrics, University Hospital of Udine, 33100 Udine, Italy; ambrogio.londero@gmail.com (A.P.L.); alicvak@gmail.com (A.N.); baccag@hotmail.it (G.B.); lorenza.driul@uniud.it (L.D.); 2 Department Medical Area, School of Medicine, University of Udine, 33100 Udine, Italy; martina.dagostin@gmail.com; 3 Endocrinology and Metabolism Unit, University Hospital of Udine, 33100 Udine, Italy; galasso.sil@gmail.com (S.G.); franco.grimaldi@asufc.sanita.fvg.it (F.G.); 4 Unit of Neonatology, University Hospital of Udine, 33100 Udine, Italy; carla.pittini@asufc.sanita.fvg.it

**Keywords:** pre-gestational diabetes, glycated haemoglobin, glycemic control

## Abstract

*Background and Objectives*: This observational study aims to determine the correlation between glycemic control with the HbA1c value and adverse obstetric outcome in women affected by pre-gestational diabetes. *Materials and Methods*: A retrospective analysis has been performed at the University Hospital of Udine. Only patients with a singleton pregnancy, pre-gestational diabetes, and known level of Hb A1c throughout pregnancy were included in the study. *Results*: According to the HbA1c level, at the beginning of pregnancy, 49 patients with HbA1c ≤ 7.0% were compared with 45 patients with HbA1c > 7.0%. Maternal age at diagnosis of the disease was significantly higher in the group with HbA1c ≤ 7% than in the group with HbA1c > 7%, 26.00 (18.00–32.00) vs. 20.00 (12.50–27.00). Women with HbA1c ≤ 7.0% reached, at term of pregnancy, significantly lower levels of HbA1c, 5.8% (5.7–6.0) vs. 6.7% (6.3–7.3). Daily insulin units were statistically different between the two groups at the end of pregnancy (47.92 (39.00–67.30) vs. 64.00 (48.00–82.00)). Proteinuria was significantly higher in the group with HbA1c > 7.0%, who delivered at earlier gestational age (37.57 (35.57–38.00) vs. 38.14 (38.00–38.43). Moreover, women with HbA1c > 7.0% had a significantly higher prevalence of an adverse composite outcome. Of note, in multivariate logistic regression analysis, pregnancy complications were significantly correlated to pre-pregnancy HbA1c > 7.0% (OR 2.95 CI.95 1.16–7.48, *p* < 0.05) independently of age, insulin treatment, and type of diabetes. *Conclusions*: Our data, obtained from a single-center cohort study, suggest that starting pregnancy with poor glycemic control might predict more complex management of diabetes in the following trimesters.

## 1. Introduction

In Italy, about 6–7% of women are affected by diabetes during pregnancy [1]. Maternal diabetes is classified as gestational diabetes, which is the most common form of diabetes during pregnancy, and pre-gestational diabetes, which includes types 1 and 2, already diagnosed before pregnancy. Type 1 pre-gestational diabetes is due to an autoimmune process that destroys the pancreatic beta-cells, which leads to onset earlier in life, the need for insulin therapy, and the potential development of vascular, renal, and neuropathic complications. In contrast, type 2 diabetes mellitus is characterized by onset later in life, peripheral insulin resistance, relative insulin deficiency, and it is associated with obesity. The incidence of both types of diabetes is increasing in childbearing women, and consequently in pregnant women [2].

During pregnancy, diabetes increases the risk of adverse perinatal outcomes such as pregnancy loss, congenital malformations, macrosomia, and preeclampsia [3]. Several studies have demonstrated that tight glycemic control might significantly improve the obstetric outcome [4]. According to the American Diabetes Association (ADA), two techniques can be used in order to evaluate the therapy efficacy: blood glucose self-monitoring (BGS) and hemoglobin A1c (HbA1c), which provides an estimate of average glucose levels over approximately 120 days [5,6]. While glucose gives immediate, day-to-day information about glycemic control, A1C gives medium to long-term information.

This observational study aims to determine the correlation between glycemic control with the HbA1c value and adverse obstetric outcome.

## 2. Materials and Methods

This study was a retrospective chart review investigation, approved by the internal review board. It was conducted following the Helsinki Declaration, and within the dictates of the general authorization to process personal data for scientific research purposes by the Italian Data Protection Authority. A total of 31,368 women delivered at the University Hospital of Udine between January 1998 and December 2017. Only patients with a singleton pregnancy, pre-gestational diabetes, and a known level of glycated hemoglobin A1c throughout pregnancy were included in the present study. The selected population had a diagnosis of types 1 or 2 diabetes mellitus before the beginning of pregnancy, or a diagnosis of pre-gestational diabetes during the current pregnancy if one of the following criteria were met at the gestational diabetes screening test: (i) fasting plasma glucose >126 mg/dL (7.0 mmol/L); (ii) random fasting plasma glucose >200 mg/dL (11.1 mmol/L); (iii) HbA1c > 6.5% (48 mmol/mol). The following data were retrieved: baseline features of patients (maternal age, maternal weight gain during pregnancy, pregravid Body Mass Index (BMI), BMI at term of pregnancy, number of spontaneous abortions, parity, race, conception mode, number of ultrasound examinations); clinical characteristics of the disease (maternal age at DM diagnosis, HbA1c at term of pregnancy, daily insulin units at the beginning and term of pregnancy, type of pre-gestational diabetes, diabetes therapy before pregnancy, history of previous gestational diabetes, first-degree familiarity for diabetes, presence of microalbuminuria, proteinuria and hypothyroidism). Pregravid data not older than 6 months before current pregnancy were considered, whereas data at the term of pregnancy were included if collected within 2 weeks after delivery. Maternal and neonatal outcomes were then retrieved, i.e., neonatal and placental weight, the prevalence of small of gestational age (SGA) and large for gestational age (LGA) neonates, delivery mode, indication for cesarean delivery, the prevalence of hypertensive disorders, premature rupture of membranes (PROM), preterm birth, urinary tract infections, Apgar score at the first and fifth minute, the prevalence of neonatal polycythemia, polyhydramnios and oligohydramnios.

A composite poor pregnancy perinatal outcome variable was considered [7,8]. This composite outcome included at least one of the following items: (1) preterm delivery (defined as delivery before 37 weeks of gestation) [9]; (2) SGA or LGA (as previously defined) [10]; (3) intrauterine fetal demise or perinatal death (as previously defined) [11,12]; (4) neonatal symptomatic hypoglycemia (as previously defined) [13]; (5) congenital malformations; (6) pregnancy-related hypertensive disorders (including pre-eclampsia) [11,14].

The analysis was performed using R (version 3.6.1—R: A language and environment for statistical computing. R Foundation for Statistical Computing, Vienna, Austria), a *p*-value < 0.05 was considered significant. The Kolmogorov–Smirnoff test assessed the normality of variables. For the principal variables considered, a descriptive statistical analysis has been carried out. For the parametric variables, a Student’s *t*-test and, for the non-parametric variables, a Wilcoxon test has been used. Concerning categorical variables, the Chi-squared test or Fisher exact test has been applied where appropriate. Afterward, univariate and multivariate logistic regression tests were performed, parsing established outcomes as dependent variables and the potential risk factors for such outcomes as independent variables. All factors with a *p*-value < 0.200 in univariate analysis were considered for multivariate analysis. Initial models included all variables and their interactions in a full factorial design. If the interactions proved to be non-significant in exploratory analysis, the model without interaction terms was adopted.

## 3. Results

Ninety-four women satisfied the inclusion criteria. This population was then subdivided into two groups depending on the median HbA1c level at the beginning of the pregnancy: 49 patients had HbA1c ≤ 7%, and 45 patients had HbA1c > 7%. The two groups were compared with each other regarding baseline features, management of pre-gestational diabetes, and obstetric outcome (Table 1). The mean maternal age at pregnancy was slightly higher in the group with HbA1c ≤ 7%, 34.00 (31.00–36.00) vs. 32.00 (28.00–37.00). Maternal weight gain during pregnancy was comparable between the two groups, 13.15 (11.00–15.35) vs. 14.00 (10.80–15.85). Pre-gestational and full-term BMI were lower in the group with HbA1c ≤ 7% than in the group with higher HbA1c. However, no statistically significant differences have been observed. Women with lower HbA1c were less frequently primigravidae, 32.65% (16/49) vs. 46.67% (21/45) and, more often, of African Race (North African 6.12% (3/49) vs. 4.44% (2/45) and Sub-Saharan African 4.08% (2/49) vs. 2.22% (1/45)). Women with higher HbA1c had a spontaneous onset of pregnancy, whereas only two women with lower HbA1c underwent an assisted reproduction. Table 2 shows the differences between the two groups throughout the course of pre-gestational diabetes during pregnancy. Maternal age at diagnosis of the disease was significantly higher in the HbA1c ≤ 7% group than in the group with HbA1c > 7%, 26.00 (18.0–32.00) vs. 20.00 (12.50–27.00). Women with HbA1c ≤ 7% compared with those with HbA1c > 7% reached, at term of pregnancy, significantly lower levels of HbA1c, 5.80 (5.70–6.00) vs. 6.70 (6.30–7.25). Daily insulin units were statistically different between the two groups at the end of pregnancy (47.92 (39.00–67.30) vs. 64.00 (48.00–82.00)), but not at the beginning, 30.00 (17.50–39.00) vs. 38.00 (24.00–44.00). In both groups, most women with pre-gestational diabetes had type 1 DM. When therapy before pregnancy was considered, no significant differences between the two groups were observed. Previous gestational diabetes and first-degree familiarity for diabetes were present in both groups without significant differences. Proteinuria was significantly higher in the group with HbA1c > 7% than in the group with HbA1c ≤7%. Moreover, microalbuminuria and hypothyroidism were more frequently present in the group with higher HbA1c. Table 3 shows the differences between the two groups in terms of their obstetric and neonatal outcomes. Women with HbA1c > 7% delivered at significantly earlier gestational ages than women with HbA1c ≤ 7%, 37.57 (35.57–38.00) vs 38.14 (38.00–38.43). No statistically significant differences were retrieved between the two groups in terms of neonatal and placental weight, but women with HbA1c > 7% tended to deliver neonates that were large in relation to their gestational age. Both groups of women had a more frequent cesarean section. At the same time, previous cesarean section was the main indication for the surgical procedure for women with HbA1c ≤ 7%, and suspected birth asphyxia was the main indication in women with HbA1c > 7%. The hypertensive disorders, the threatened preterm labor and the urinary tract infections were more common in the group with high rather than low HbA1c. As for the neonatal outcome, no significant differences were found between the groups, but women with HbA1c > 7% had a much higher prevalence of adverse composite outcomes.

In multivariate logistic regression analysis, pregnancy complications (composite outcome) were significantly correlated to pre-pregnancy HbA1c > 7% (OR 2.95 CI.95 1.16–7.48, *p* < 0.05) independently of age, insulin treatment, and type of diabetes. Table 4 shows the univariate and multivariate logistic regression analysis considering each component of the composite outcome. Moreover, a high pre-pregnancy HbA1c was found to be significantly correlated with younger age at the time of pregnancy (OR 2.98 CI. 95 1.23–7.24, *p* < 0.05) independently of the type of diabetes as well as the younger age at diabetes diagnosis (OR 2.69 CI.95 0.93–7.79, *p* = 0.069; not significant because of 25 missing values). Accordingly, a high term pregnancy HbA1c was shown to be correlated with high pre-pregnancy HbA1c (OR 4.88 CI.95 1.73–13.75, *p* < 0.05) independently of the age at pregnancy and amount of insulin treatment. Among type I diabetes, proteinuria during pregnancy was significantly correlated with the amount of insulin used at the beginning of pregnancy (OR 1.09 CI.95 1.00–1.19, *p* < 0.05) independently of age, pre-pregnancy HbA1c, and the amount of insulin therapy at pregnancy term. Furthermore, preterm delivery was associated with preeclampsia, oligohydramnios, and SGA (OR 1.57 CI 1.34–1.85, *p* < 0.05) independently of age, pre-pregnancy BMI, nulliparity, and pre-pregnancy HBA1c.

## 4. Discussion

This study has demonstrated that patients with HbA1c >7% at the beginning of pregnancy were significantly younger and had received the diagnosis of diabetes significantly earlier in life than patients with Hb1Ac ≤ 7%. Moreover, at pregnancy term, these patients had a significantly higher level of HbA1c, even though they were given much more insulin during pregnancy and had more frequently high proteinuria. The study also shows that patients with HbA1c > 7% had a poorer composite pregnancy outcome and delivered significantly earlier than women with HbA1c ≤ 7%.

A1c is created by the glycation of the N-terminus of the hemoglobin beta chain [15]. The hemoglobin in reticulocytes has minimal A1c content, but as hemoglobin continuously reacts with glucose, A1c gradually accumulates during the erythrocyte life span, depending on the mean blood glucose level [16]. For this reason, A1C gives a reliable estimate of the average glycemic exposure of the previous 12 weeks.

It is well recognized that with A1c < 6–6.5%, women with diabetes have the lowest rates of adverse perinatal outcomes [4,5,17]. According to ADA recommendations, the HbA1c target in pregnancy is 6–6.5%, but the target may be relaxed to ≤ 7%, if necessary, to prevent hypoglycemia [5].

Our data have shown that maternal age at diagnosis of the disease was significantly lower in the group with HbA1c > 7%, thus confirming that the disease is much more severe when acting over a long period. Another plausible hypothesis is that older women were motivated to optimally control their plasma glucose levels to achieve pregnancy, while younger women mainly focused on pregnancy seeking, regardless of their metabolic control. This point may reflect how the local campaign has failed to prepare, especially young diabetic women during the preconception care. A meta-analysis and systematic review have proved the effectiveness of pre-pregnancy care in reducing congenital malformation, perinatal mortality, and lower maternal HbA1c in the first trimester of pregnancy [18]. The most extensive contemporary cohort study has clearly shown that women with type 2 diabetes are less likely to attend the pre-pregnancy programs, maybe because type 2 diabetes is, wrongly, still considered less severe than type 1 diabetes [19]. These authors also observed how younger women with type 1 diabetes (aged 15–24 years) were most at risk of starting pregnancy with high HbA1c, recommending that contraception awareness be improved, especially among pediatric and young adult diabetic populations [19].

While the role of the sub-fraction A1c glycated hemoglobin as a biomarker of the recent exposure to hyperglycemia is well-established [20], our data suggest that it may reflect a chronic trend of pre-gestational diabetes. Therefore, starting pregnancy with poor glycemic control might predict more complex management of diabetes in the following trimesters. This study’s data further confirm this observation, showing a significantly poorer composite obstetric outcome in women with HbA1c > 7%. Consequently, the likelihood of pregnancy complications could be expected to be higher in women with poor control of the disease at the beginning of gestation. It might, indeed, be speculated that the longer the exposition to the disease, the poorer the glycemic control throughout pregnancy, which highlights the need for much more insulin therapy and a higher risk of complications of diabetes in the mother and the fetus.

Our study showed that women with HbA1c over 7% were inclined to deliver LGA neonates. Previous studies [21] have demonstrated that LGA birth weight is the most common complication of pregnancy in women with pre-gestational diabetes. A recent national cohort study [19], including 15,290 women with pre-existing diabetes, supported the association between LGA birth weight and higher HbA1c. However, the same study found no association of LGA with maternal obesity and deterioration in maternal glycemia [19]. Furthermore, women affected by pregestational diabetes and not treated for hypertension or hyperlipidemia were at increased risk of growing LGA neonates, thus suggesting that these factors could affect the placental function and impact fetal growth patterns [19]. Our finding lacks statistical significance, perhaps because A1c does not adequately capture postprandial hyperglycemia, which is directly correlated with fetal overgrowth [22]. Several studies have demonstrated that intermittent hyperglycemia (usually associated with normal HbA1c) is more correlated with accelerated fetal growth than chronic hyperglycemia (usually associated with higher HbA1c) [23]. Therefore, HbA1c is not the best biomarker for predicting fetal macrosomia.

The cesarean delivery rate was comparable for both groups. However, among women with HbA1c > 7%, the main indication for cesarean delivery was the non-reassuring fetal status. Considering that these women delivered at significantly earlier gestational age, it could be contended that they often underwent labor induction because of pregnancy complications due to diabetes. Labor induction is associated with a prolonged fetal surveillance period, which extends from cervical maturation to the second stage of labor, leading to a higher likelihood of registering electronic fetal monitoring abnormalities and a consequent higher obstetrical intervention. Nevertheless, there could be another explanation for this phenomenon. Since fetal tolerance to repeated hypoxemia in labor is influenced by fetal anaerobic reserve and overall pre-labor health, it could be speculated that fetuses of diabetic mothers, chronically exposed to poor glycemic control, decompensate easier during labor, thus increasing the cesarean delivery rate [24].

According to our data, women with HbA1c > 7% tend to deliver significantly earlier (35.57–38.00) than women with HbA1c ≤ 7% (38.00–38.43). Several factors could explain the higher rate of preterm birth in women with HbA1c, including the high trend of iatrogenic birth at 35–36 weeks in the presence of both an altered glycemic diary and ultrasound signs of metabolic failure, or the increased rate of hypertensive disorders and preeclampsia [25].

Most studies on this topic in the literature have compared type 1 versus type 2 diabetes, showing a worsening of diabetes-related complications during pregnancy, reporting a trend of higher maternal glucose levels, higher rates of preterm births, and of LGA babies in women with type 1 diabetes compared with type 2 diabetes [2,26]. By contrast, women with type 2 diabetes tend to be older, have higher rates of obesity, greater ethnic diversity, and greater socioeconomic deprivation [21,27,28,29]. Interestingly, it has been observed that there is a poor correlation between HbA1c and perinatal complications in women with type 2 diabetes, thus suggesting that other factors, such as obesity and insulin resistance, may influence the gestational outcomes more than the glycemic control [19,30]. The low number of women with type 2 diabetes in our population does not allow us to address this interesting issue.

The present study has several limitations. A criticism usually levelled against observational studies is that they may suffer from the effect of bias. Usually, the primary source of bias in a cohort study is patient selection. In the present study, this bias was considerably appeased by the fact that patients encountered, overall, the same staff sharing the same treatment protocols. Another limitation lies in the nature of the hospital center specialized in the advanced care of patients. It might be possible that the high-risk profile of the background population worsens the overall outcome of the cohort studied. However, there are also some strengths to be recognized. First of all, this study provides information about perinatal outcomes in women affected by a relatively rare condition from a single hospital center in Northeast Italy. Secondly, as only one hospital center was involved, women affected were followed up and treated uniformly, which guarantees an important reduction in possible bias related to management.

Taken together, these findings could have some relevance for daily clinical practice. Pregnancy is a critical challenge for women affected by pre-gestational diabetes in unsatisfactory control. The fact that these women reach the term of gestation with proteinuria and even higher glycated hemoglobin levels may reflect the disease progression during pregnancy with the onset of organ damage. Therefore, health care providers should appropriately manage these patients in the post-partum period, as the disease could have a new clinical presentation. Finally, these data indicate the need for effective interventions that improve metabolic control during pregnancy to decrease the rate of short and long-term complications.

## 5. Conclusions

This study demonstrates a correlation between HbA1c over 7% at the beginning of pregnancy and adverse maternal outcomes. The explanation is that starting pregnancy with poor glycemic control makes the management of diabetes more difficult in the following trimesters.

## Figures and Tables

**Table 1:** Baseline features of patients affected by pregestational diabetes who delivered at University Hospital of Udine between 1998 and 2017 and subdivided in two groups: 49 with HbA1c higher than 7% and 45 with HbA1c lower than 7%.

	HbA1c ≤ 7.0% (49)	HbA1c > 7.0% (45)	*p*
Maternal age at pregnancy (years)	34.00 (31.00–36.00)	32.00 (28.00–37.00)	0.082
Weight gain during pregnancy (kg)	13.15 (11.00–15.35)	14.00 (10.80–15.85)	0.499
Pre-gestational BMI (kg/m^2^)	23.40 (22.00–26.00)	24.00 (21.90–28.00)	0.764
BMI at term of pregnancy (kg/m^2^)	28.40 (26.50–31.00)	30.00 (27.00–33.00)	0.324
Spontaneous miscarriages	0.00 (0.00–1.00)	0.00 (0.00–0.00)	<0.05
Primigravidas	32.65% (16/49)	46.67% (21/45)	0.165
Race			
Caucasian	89.80% (44/49)	93.33% (42/45)	0.539
North-African	6.12% (3/49)	4.44% (2/45)	0.717
Sub-Saharan African	4.08% (2/49)	2.22% (1/45)	0.608
Conception mode			
IVF/ET	2.04% (1/49)	0.00% (0/45)	0.335
IUI	2.04% (1/49)	0.00% (0/45)	0.335
Spontaneous	95.92% (47/49)	100.00% (45/45)	0.171
Number of ultrasound examinations during pregnancy	6.50 (4.00–8.25)	8.00 (7.00–10.00)	0.063

**Table 2:** Differences between the two groups of women with pre-gestational diabetes throughout the course of the disease during pregnancy.

	HbA1c ≤ 7% (49)	HbA1c > 7% (45)	*p*
Maternal age at DM diagnosis (years)	26.00 (18.00–32.00)	20.00 (12.50–27.00)	<0.05
HBA1c (%) at term of pregnancy	5.8 (5.7–6.0)	6.7 (6.3–7.3)	<0.05
Daily insulin units (at start of pregnancy)	30.00 (17.50–39.00)	38.00 (24.00–44.00)	0.059
Daily insulin units (at term of pregnancy)	47.92 (39.00–67.30)	64.00 (48.00–82.00)	<0.05
Pre-gestational diabetes type			
Type 1	75.51% (37/49)	82.22% (37/45)	0.427
Type 2	24.49% (12/49)	17.78% (8/45)	0.427
Diabetes therapy before pregnancy			
Diet/no therapy	18.37% (9/49)	11.11% (5/45)	0.324
Metformin	6.12% (3/49)	6.67% (3/45)	0.914
Multi-injective Insulin	51.02% (25/49)	60.00% (27/45)	0.382
Pump	24.49% (12/49)	22.22% (10/45)	0.795
Previous GDM	12.24% (6/49)	6.67% (3/45)	0.359
First degree familiarity for diabetes	85.71% (42/49)	80.00% (36/45)	0.461
Microalbuminuria	2.04% (1/49)	8.89% (4/45)	0.139
Proteinuria	4.08% (2/49)	20.00% (9/45)	<0.05
Hypothyroidism	20.41% (10/49)	20.00% (9/45)	0.961

**Table 3:** Obstetric and neonatal outcomes of patients with pregestational diabetes. Differences between women with HbA1c ≤7% and women with HbA1c >7%.

	HbA1c ≤7% (49)	HbA1c >7% (45)	*p*
Gestational age at delivery (weeks)	38.14 (38.00–38.43)	37.57 (35.57–38.00)	<0.05
Neonatal weight (gr)	3484.00 (3140.00–3710.00)	3357.50 (3048.00–3832.00)	0.882
Placental weight (gr)	652.00 (552.50–777.50)	570.00 (500.00–650.00)	0.145
SGA < 10° percentile	0.00% (0/45)	2.38% (1/42) (**)	0.298
LGA > 90° percentile	35.56% (16/45)	47.62% (20/42)	0.254
LGA > 97° percentile	24.44% (11/45)	40.48% (17/42)	0.110
Delivery mode			
Vaginal delivery	28.57% (14/49)	20.00% (9/45)	0.334
Vaginal operative delivery	16.33% (8/49)	17.78% (8/45)	0.852
Cesarean delivery	55.10% (27/49)	62.22% (28/45)	0.484
Indications for cesarean delivery			
Previous cesarean delivery	40.74% (11/27)	21.43% (6/28)	0.121
Suspected birth asphyxia	22.22% (6/27)	28.57% (8/28)	0.589
Maternal indications	11.11% (3/27)	17.86% (5/28)	0.478
Labor dystocia (mechanical or dynamic)	11.11% (3/27)	21.43% (6/28)	0.301
Fetal malpresentation	11.11% (3/27)	3.57% (1/28)	0.282
Abnormal fetal growth/other	3.70% (1/27)	7.14% (2/28)	0.574
Composite pregnancy negative outcome (*)	40.82% (20/49)	71.11% (32/45)	<0.05
Hypertensive disorders associated with pregnancy	10.20% (5/49)	24.44% (11/45)	0.066
Preeclampsia	4.08% (2/49)	15.56% (7/45)	0.059
Hypertension	10.20% (5/49)	24.44% (11/45)	0.066
HELLP	0.00% (0/49)	2.22% (1/45)	0.294
Threatened preterm labor	10.20% (5/49)	20.00% (9/45)	0.183
Urinary tract infection	2.04% (1/49)	8.89% (4/45)	0.139
Neonatal gender			
F	56.82% (25/44)	47.62% (20/42)	0.393
M	43.18% (19/44)	52.38% (22/42)	0.393
Apgar score 1° minute	8.00 (7.00–8.00)	8.00 (6.00–8.75)	0.637
Apgar score 5° minute	9.00 (9.00–9.00)	9.00 (8.00–9.00)	0.205
PROM	8.16% (4/49)	6.67% (3/45)	0.782
NICU hospitalization	10.20% (5/49)	20.00% (9/45)	0.183
Neonatal Polycitemia	0.00% (0/49)	2.22% (1/45)	0.294
Polihydramnios	0.00% (0/49)	4.44% (2/45)	0.136
Oligohydramnios	0.00% (0/49)	4.44% (2/45)	0.136

(**) Only one SGA < 3° percentile. (*) composite outcome included at least one of the following items: (1) preterm delivery (<37 weeks of gestation); (2) SGA or LGA; (3) intrauterine fetal demise or perinatal death; (4) neonatal symptomatic hypoglycemia; (5) congenital malformations; (6) pregnancy-related hypertensive disorders (including pre-eclampsia).

**Table 4:** Univariate and multivariate ((**) or (ŧ)) logistic regression analysis considering, as dependent variables, the composite outcome and the separated components of the composite outcome.

	OR (CI.95)	*p*	aOR (CI.95)	*p*
Composite outcome				
Pre-pregnancy HbA1c > 7.0%	3.57 (1.51–8.44)	<0.05	2.95 (1.16–7.48)	<0.05 (**)
Components of the composite outcome				
(1) Preterm delivery (<37 weeks’ gestation)				
Pre-pregnancy HbA1c > 7.0%	4.78 (1.69–13.54)	<0.05	4.93 (1.66–14.66)	<0.05 (ŧ)
(2) SGA or LGA				
Pre-pregnancy HbA1c > 7.0%	1.81 (0.77–4.28)	0.175	1.99 (0.82–4.84)	0.130 (ŧ)
(3) Intrauterine fetal demise or perinatal death				
Pre-pregnancy HbA1c > 7.0% (*)	--	--	--	--
(4) Neonatal symptomatic hypoglycemia				
Pre-pregnancy HbA1c > 7.0% (*)	--	--	--	--
(5) Congenital malformations				
Pre-pregnancy HbA1c > 7.0% (*)	--	--	--	--
(6) Pregnancy-related hypertensive disorders				
Pre-pregnancy HbA1c > 7.0%	2.85 (0.90–8.97)	0.074	3.04 (0.94–9.77)	0.062 (ŧ)

(*) No cases registered. (**) Multivariate logistic regression with adjustment for age, insulin treatment, and type of diabetes. (ŧ) Multivariate logistic regression with adjustment for age. Acronyms: OR = odds ratio; aOR = adjusted odds ratio.

## Data Availability

The data that support the findings of this study are available, but restrictions apply to the availability of these data, which were used under license for the current study, and so are not publicly available. Data are, however, available from the authors upon reasonable request and with the permission of the Internal Review Board.
